# Gene regulation in activated microglia by adenosine A_3_ receptor agonists: a transcriptomics study

**DOI:** 10.1007/s11302-022-09916-9

**Published:** 2023-01-27

**Authors:** Alejandro Lillo, Joan Serrano-Marín, Jaume Lillo, Iu Raïch, Gemma Navarro, Rafael Franco

**Affiliations:** 1 Department of Biochemistry and Physiology, School of Pharmacy and Food Science, Universitat de Barcelona, Barcelona, Spain; 2 CiberNed, Network Center for Neurodegenerative Diseases, National Spanish Health Institute Carlos III, Madrid, Spain; 3 Molecular Neurobiology laboratory, Department of Biochemistry and Molecular Biomedicine, Faculty of Biology, Universitat de Barcelona, Barcelona, Spain; 4 Institute of Neurosciences, Universitat de Barcelona, Barcelona, Spain; 5 School of Chemistry, Universitat de Barcelona, Barcelona, Spain

**Keywords:** Alzheimer’s disease, Parkinson’s disease, Neurodegeneration, Adenosine, Receptors, Microglia, Neuroinflammation

## Abstract

Most neurodegenerative disorders, including the two most common, Alzheimer’s disease (AD) and Parkinson’s disease (AD), course with activation of microglia, the resident innate immune cells of the central nervous system. A_3_ adenosine receptor (A_3_R) agonists have been proposed to be neuroprotective by regulating the phenotype of activated microglia. RNAseq was performed using samples isolated from lipopolysaccharide/interferon-γ activated microglia treated with 2-Cl-IB-MECA, a selective A_3_R agonist. The results showed that the number of negatively regulated genes in the presence of 2-Cl-IB-MECA was greater than the number of positively regulated genes. Gene ontology enrichment analysis showed regulation of genes participating in several cell processes, including those involved in immune-related events. Analysis of known and predicted protein-protein interactions showed that Smad3 and Sp1 are transcription factors whose genes are regulated by A_3_R activation. Under the conditions of cell activation and agonist treatment regimen, 2-Cl-IB-MECA did not lead to any tendency to favor the expression of genes related to neuroprotective microglia (M2).

**Supplementary Information:**

The online version contains supplementary material available at 10.1007/s11302-022-09916-9.

## Introduction

### The inflammatory component in neurodegenerative diseases

Age is the main risk factor in neurodegenerative disorders, which are the cause of approximately 6.8 million deaths each year (https://www.who.int/news/item/27-02-2007-neurological-disorders-affect-millions-globally-who-report; accessed on September 20, 2022). Alzheimer’s disease (AD) and Parkinson’s disease (PD) are the two most prevalent. Common features of neurodegenerative diseases include deposition of protein aggregates, progressive neuronal death (cortical and hippocampal neurons in AD, and nigrostriatal dopaminergic neurons in PD), and expression of activation markers in microglia surrounding neurodegenerative structures. The mechanism of neuronal death is still under intense investigation; on the one hand, factors such as oxidative stress, mitochondrial dysfunction, and glutamate excitotoxicity are attributed to it. Neuroprotective approaches generally target receptors on neurons. Several preclinical studies have suggested, over the years, that neuroprotection can be afforded via modulation of the functionality of adenosine receptors expressed in neurons [1–3]. Four are the receptors for the autocoid adenosine, A_1_, A_2A_, A_2B_, and A_3_; they all belong to the G-protein-coupled receptor (GPCR) superfamily and are widely distributed in the mammalian body. The A_2A_ and the A_2B_ are coupled to G_s_, and their activation leads to increases in intracellular cAMP, whereas the A_1_ and the A_3_ are coupled to G_i_ and their activation leads to decreases in intracellular cAMP. In the last two decades, the adenosinergic system, and particularly the A_2A_R, has gained relevance mainly due to two factors. A first in class A_2A_R antagonist, istradefylline, has been approved in Japan and the USA for the adjuvant therapy of patients with PD. This development has raised hopes in Academia and in the Pharmaceutical Industry that there will be more drug approvals targeting adenosine receptors for a wide range of diseases, from obesity to cancer [4–10].

As suffering neurons may not be able to expand their life on their own, there is the alternative of targeting receptors on glial cells. In diseases that occur with neuroinflammation, microglia acquire interest. In fact, microglial activation, previously considered harmful, is now becoming relevant due to the possibility of directing these cells towards a neuroprotective phenotype [11–15]. The nomenclature: M0 for resting, M1 for pro-inflammatory, and M2 for neuroprotective are used as a first approximation to understand the phenotypic status of a microglial cell in a given environment [16]. Based on the expression of cannabinoid receptors in activated microglia and in primary cultures of a transgenic model of Alzheimer’s disease (APP_Sw,Ind_), it was hypothesized that microglia help to delay cognitive decline by several months. In fact, these animals, which overexpress the Swedish and Indiana mutated form of the human amyloid precursor protein, do not present gross alterations until later in life and among the few differential trends with control animals is a “pre-activated” phenotype in the microglia isolated from 3-day-old pups [17].

A_3_R agonists have shown promising anti-inflammatory effects in preclinical assays. They have been considered for the therapy of serious diseases coursing with marked immune system dysfunction, such as rheumatoid arthritis and psoriasis [18], among others. Also, A_3_R agonists have been suggested as having potential to combat retinal neurodegeneration [19], post-ischemic brain damage [20], or neuroinflammation after subarachnoid hemorrhage [21]. Even though there are several pharmacological and functional studies related to the adenosinergic system, the effect of extracellular adenosine on gene expression is poorly understood. Here, we aimed to determine by a transcriptomic approach whether (i) activation of the A_3_R in activated microglia modulates gene expression, with special attention to genes encoding factors involved in immune responses, and (ii) microglia are biased towards the neuroprotective phenotype.

## Methodology

### Reagents

Trizol (Ambion Life Technologies, 15696026), chloroform (Sigma_Aldrich, C2432-500ML), isopropanol (PanReac AppliChem, 131090.1211), lipopolysaccharide (Sigma_Aldrich, L4391-1MG), interferon-γ human (Sigma Aldrich; I3265-1MG), 2-chloro-N6-(3-iodobenzyl)-adenosine-5'-N-methyluronamide (2-Cl-IB-MECA; ToCris Bioscience; 1104).

### Isolation and activation of microglia

To prepare primary microglia, the brain of C57BL/6J wild-type mice was removed at postnatal days 2 to 4. Microglial cells were isolated and plated at a confluence of 40,000 cells/0.32 cm^2^. Briefly, the tissue was dissected, and after carefully removing the meninges, the brain samples were digested with 0.25% trypsin for 20 min at 37 °C. Trypsinization was stopped by adding an equal volume of culture medium (Dulbecco’s modified Eagle medium-F-12 nutrient mixture, Invitrogen). Cells were brought to a single cell suspension by repeated pipetting followed by passage through a 100-μm pore mesh. Pelleted (7 min, 200 g) cells were resuspended in supplemented DMEM and seeded at a density of 3.5 × 10^5^ cells/mL in 6-well plates. Twenty four hours later, the medium was replaced by Dulbecco’s modified Eagle medium supplemented with 2 mM L-glutamine, 100 U/mL penicillin/streptomycin, MEM Non-Essential Amino Acid Solution (1/100) and 10% (v/v) heat inactivated Fetal Bovine Serum (FBS) (all supplements were from Invitrogen, Paisley, Scotland, United Kingdom). Cells were maintained in a humid atmosphere of 5% CO_2_ at 37 °C and were used for RNA extraction after 15 days of culture. Cells were activated for 48 h with 0.01% (v/v) LPS and 0.002% (v/v) IFN-γ in Dulbecco’s modified Eagle medium and, at 24, 32, and 40 h, vehicle or 200 nM 2-Cl-IB-MECA was added.

After this, we proceeded to extraction of total RNA with the Trizol-based method using chloroform and isopropanol to purify the RNA. Quality control was assessed by measuring the 280/260 absorption ratio and the RIN in the services of the University of Barcelona (CCiTUB: Scientific and Technological Centers of the Universitat de Barcelona). Sequencing was done in the German facilities of Novogene using the following protocol: Messenger RNA was purified from total RNA using poly-T oligo-attached magnetic beads. After fragmentation, the first-strand cDNA was synthesized using random hexamer primers, followed by the second-strand cDNA synthesis using either dUTP for directional library or dTTP for non-directional library. For the non-directional library, it was ready after end repair, A-tailing, adapter ligation, size selection, amplification, and purification. For the directional library, it was ready after end repair, A-tailing, adapter ligation, size selection, USER enzyme digestion, amplification, and purification.

The library was checked with Qubit and real-time PCR for quantification and bioanalyzer for size distribution detection. Quantified libraries are pooled and sequenced on Illumina platforms, according to effective library concentration and data amount. Novogene NovaSeq 6000 was the platform, pair-end 150 (PE150) the sequencing strategy, and 151+8+8+151 the sequencing cycles.

The clustering of the index-coded samples was performed according to the manufacturer’s instructions. After cluster generation, the library preparations were sequenced on an Illumina platform, and paired-end reads were generated.

For quality control, raw data (raw reads) in “fastq” format were firstly processed through *Perl *scripts. In this step, clean data (clean reads) were obtained by removing, from raw data, the reads containing adapter, reads containing poly-N, and low-quality reads. At the same time, Quality scores, Q20 and Q30, and GC content were also considered. All the downstream analyses were done using the clean data.

To map the reads, reference genome and gene annotation files were downloaded from the *Ensembl* genome browser. Hisat2 v2.0.5 was used for both indexing and alignment of clean paired-end reads to the reference genome. We selected Hisat2 as the mapping tool because it can generate a database of splice junctions based on the gene model annotation file. Therefore it provides better mapping results than other non-splice mapping tools.

### RNAseq data processing

Feature counts v1.5.0-p3 software was used to count the reads numbers mapped to each gene. And then fragments per kilobase of each gene was calculated based on the length of the gene and reads count mapped to this gene. The expected number of fragments per kilobase of transcript sequence per millions base pairs sequenced (FPKM) considers the effect of sequencing depth and gene length for the reads count at the same time and is currently the most commonly used method for estimating gene expression levels. 

### Differential expression analysis

Differential expression analysis of two conditions/groups (three biological replicates per condition) was performed using the DESeq2R package (1.20.0). DESeq2 with biological replicates provides statistical means for determining differential expression in gene expression data using a model based on the negative binomial distribution. The resulting *P* values were adjusted using the Benjamini and Hochberg’s approach for controlling the false discovery rate. Prior to differential gene expression analysis, for each sequenced library, the read counts were adjusted by edgeR program package through one scaling normalized factor. Differential expression analysis of two conditions was performed using the “edgeR R” package (version 3.22.5).

Only those genes whose expression comparison had a false discovery rate (FDR) < 0.05, and a fold change (FC) > |1.5| were selected and classified into those whose expression was upregulated or downregulated in each specific treatment.

### Gene set enrichment analyses

The STRING online tool (defined as a “*database of known and predicted protein-protein interactions*” https://string-db.org/ accessed on April 15, 2022) was used for obtaining the GOs of the DEGs whose expression decreased (downregulated) or increased (upregulated) with the following settings: no additional shells (only provided proteins were considered), medium confidence (0.4), and full STRING network, which includes direct (physical) and indirect (functional) interactions. These analyses provide a list of overrepresented GOs in each group of DEGs (those corresponding to the genes with increased expression, and those corresponding to the genes with decreased expression). Then, these GOs were clustered using the REVIGO online tool (http://revigo.irb.hr/), which reduces the number of variables and makes possible to group the GOs by similarity in a 2D plot. Finally, for improving the informative capacity of the resulting graphs, the Cytoscape software (v.3.9.1) (https://cytoscape.org/) was used.

Also, the Enrichr online tool “*interactive and collaborative HTML5 gene list enrichment analysis tool*” (https://maayanlab.cloud/Enrichr/ accessed on Abril 30, 2022) was used, to assess which transcription factors (TFs) were overrepresented; of the different options offered within Enrichr, we selected TRRUST (Transcriptional Regulatory Relationships Unraveled by Sentence-based Text-mining v.2), which is a manually curated database of transcriptional regulatory pathways and TFs. Afterwards, the STRING online tool was used again for assessing for different interactions between the overrepresented TFs. The STRING settings were the same described in the previous paragraph.

## Results

### RNAseq in activated microglia in the presence of an agonist of the A_3_R

The aim of the article was not to assess how adenosine receptors might affect microglial activation but rather how an A_3_R agonist might affect the phenotype of activated microglia. Accordingly, we designed an experiment in which microglial cells were activated using a standard protocol, i.e., using LPS and IFN-γ and leaving the cells for 48 h before obtaining RNA. Comparison of differentially expressed genes was performed on total RNA isolated from activated primary microglia treated or not with 2-Cl-IB-MECA. To be sure that the A_3_R agonist would be able to regulate gene transcription, we thought that (i) at least 8 h after addition of the agonist was required for detecting gene activation/repression and that (ii) various doses, spaced over time, were required to mimic the continuous presence of adenosine in the extracellular milieu when microglia are activated in a physiological setting.

**Fig. 1:**
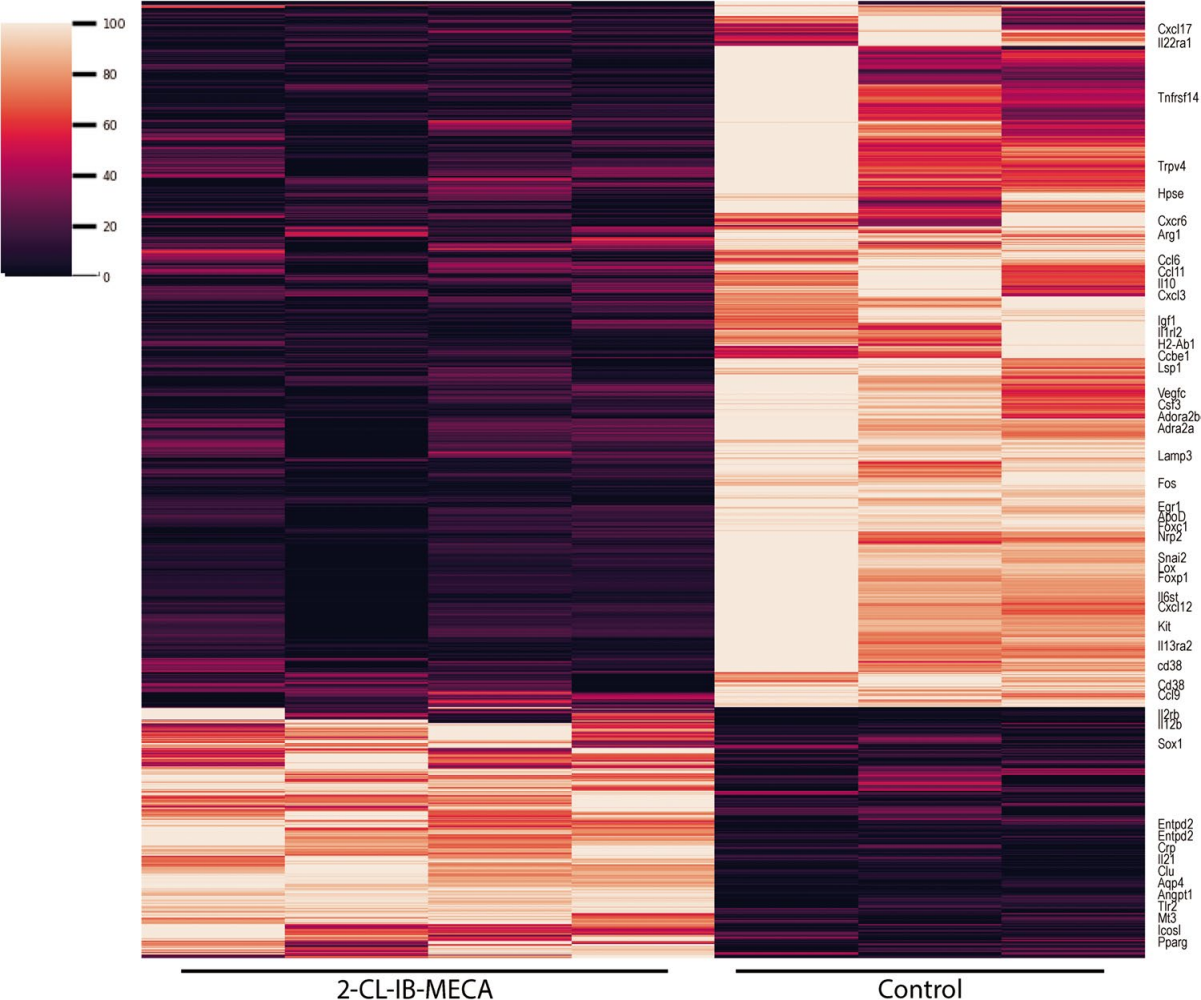
Heatmap of the differentially expressed genes in activated microglia. Columns indicate the sample (quadruplicates for each condition: control in the 4 columns on the right and treated with 2-Cl-IB-MECA in the 4 columns on the left). Only the name of genes related to inflammation and pathways related to cytokines are shown. The heat map shows only those genes that, when comparing control and agonist treatment, met the criteria: FDR < 0.05 and FC > |1.5|. Darker colors indicate downregulated gene expression in microglia treated with 2-Cl-IB-MECA, while lighter colors indicate upregulated gene expression

RNA isolation was carried out 48 h after microglial activation and 2-Cl-IB-MECA was added to the culture at 24, 32, and 40 h. mRNA sequencing and counts were performed as indicated in methods. Data analysis showed a large number of differentially expressed genes when activated microglia were treated with 2-Cl-IB-MECA. Comparing the two conditions (plus/minus A_3_R agonist) and with these two criteria: false discovery rate (FDR) < 0.05 and fold change (FC) > |1.5|, the number of genes whose expression was downregulated by agonist treatment (1414) was greater than the number of genes whose expression was upregulated (502). The heat maps are shown in Fig. 1 (see complementary information in supplementary Table S1).

**Fig. 2:**
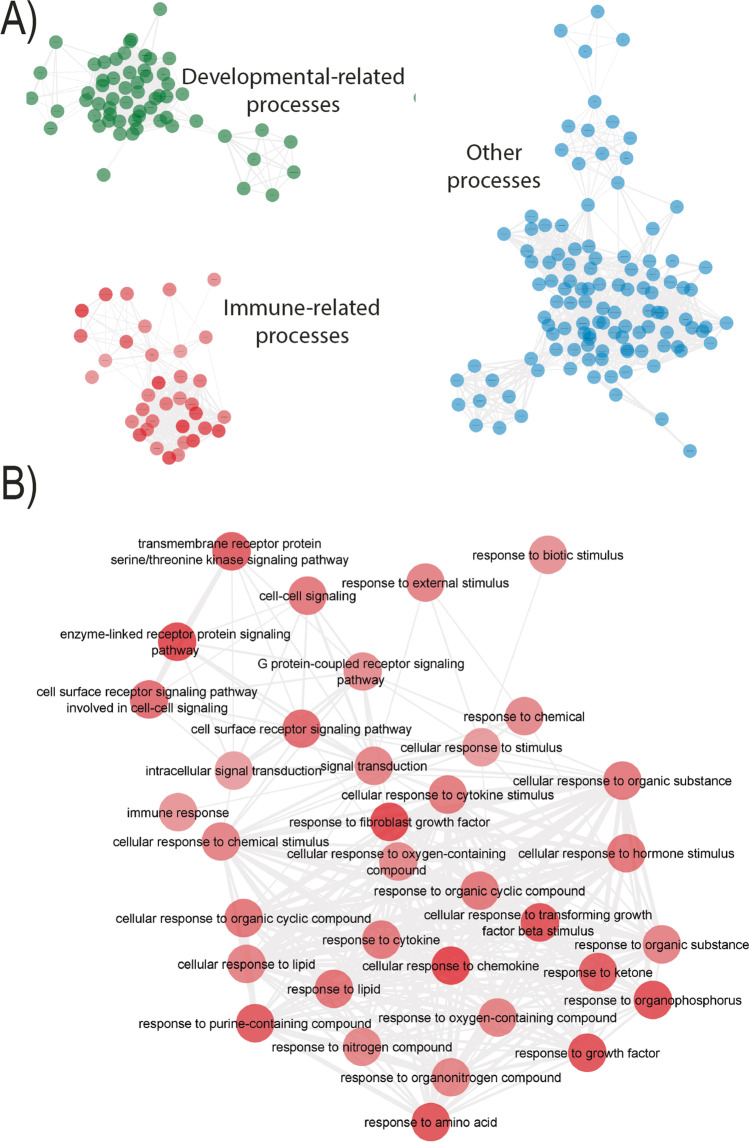
Microglial events that are regulated by transcription factors whose gene expression is downregulated by treatment with 2-Cl-IB-MECA, the receptor agonist. **A** 3 clusters reflecting processes in which genes downregulated upon 2-Cl-IB-MECA treatment are involved. **B** Immune-related microglial events that are regulated by the genes are downregulated by 2-Cl-IB-MECA treatment

The gene set enrichment analysis (GSEA) was subsequently performed taking into account the genes whose expression was downregulated genes upon A_3_R agonist treatment. When only downregulated gene expression for transcription factors were considered 3, clusters appeared (Fig. 2A). In fact, using the genes whose expression decreases, several (145) gene ontologies (GOs) were found (Fig. 2). They were mainly clustered within developmental-related and immune-related processes. The rest corresponded to GOs from cell cycle control to regulation of cell adhesion and cell-to-cell communication regulation. Figure 2B shows the detail of the several GOs altered upon A_3_R agonist activation within immunological-related events. Table 1 shows GOs when genes with increased expression were considered and only those that were significant (FDR < 0.05). The most significant were those concerning regionalization and pattern specification.

**Table 1:** Gene ontologies overrepresented when considering genes whose expression was increased upon 2-Cl-IB-MECA treatment

GO ID	Term description	Observed gene count	Background gene count	Strength	False discovery rate
GO:0003002	Regionalization	24	348	0.65	4.32e-05
GO:0007389	Pattern specification process	27	445	0.59	4.32e-05
GO:0009952	Anterior/posterior pattern specification	18	222	0.72	0.00014
GO:0009653	Anatomical structure morphogenesis	68	2244	0.29	0.00025
GO:0009954	Proximal/distal pattern formation	8	37	1.15	0.00091
GO:0048731	System development	105	4350	0.19	0.0021
GO:0032501	Multicellular organismal process	138	6272	0.15	0.0028
GO:0007275	Multicellular organism development	113	4921	0.17	0.0058
GO:0032502	Developmental process	125	5629	0.16	0.0064
GO:0048856	Anatomical structure development	118	5258	0.16	0.0077
GO:0048704	Embryonic skeletal system morphogenesis	10	105	0.79	0.0125
GO:0048706	Embryonic skeletal system development	11	139	0.71	0.0212
GO:0006811	Ion transport	38	1214	0.31	0.0418

**Fig. 3:**
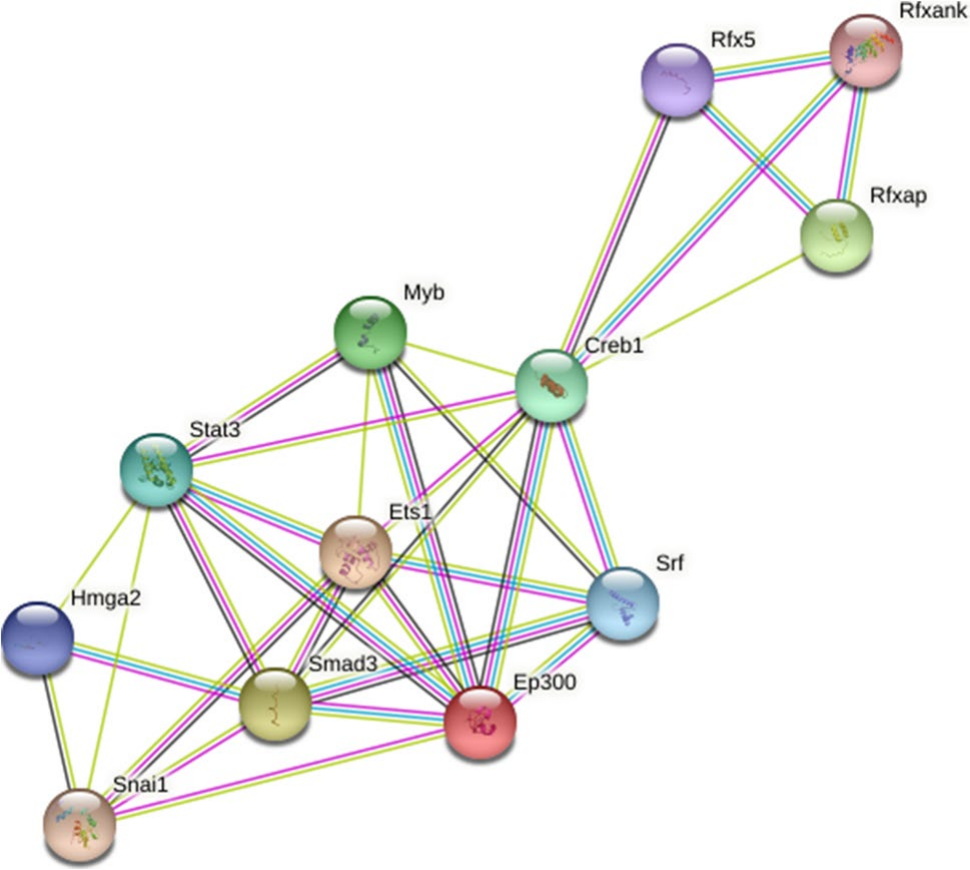
STRING analysis of interactions between the product of genes whose expression is decreased upon A_3_R agonist treatment. The colors of the connection lines represent the different types of making associations, either from known or predicted interactions: from text mining (lime), experimentally determined (magenta), gene co-occurrence (blue), and co-expression (black). Creb1, cAMP-responsive element-binding protein 1; Ets1, ETS proto-oncogene 1; Smad3, SMAD family member 3; Ep300, E1A-binding protein p300; Srf, serum response factor; Rfx5, regulatory factor X5; Rfxank, regulatory factor X associated ankyrin-containing protein; Rfxap, regulatory factor X associated protein; Stat3, signal transducer and activator of transcription 3; Hmga2, high-mobility group AT-hook 2; Snai1, snail family transcriptional repressor 1; Myb, myeloblastosis family of transcription factors

Analysis of known and predicted interactions, taking into account the protein products of the downregulated genes, reveals cAMP-responsive element-binding protein 1 (Creb1) as a node that connects two networks. One of the networks contains the regulatory factor X and associated proteins, and in the other network, the proto-oncogene ETS 1 (Ets1) and the member of the SMAD family 3 (Smad3) stand out (Fig. 3).

We finally addressed the expression of genes for biomarkers of M1 and M2 microglia. Treatment of activated microglia with 2-Cl-IB-MECA led to both upregulation and downregulation of the expression of pro-inflammatory genes, with the chemokine ligand CXCL11 being the product of the gene whose expression was most increased and the chemokine ligand CCL19 being the product of the gene whose expression decreased in greater magnitude. Following treatment with the A_3_R agonist, the expression of several genes encoding M2 biomarkers was decreased with the exception of the peroxisome proliferator-activated receptor γ (PPAR-γ) gene, whose expression was increased. Especially relevant was the decrease in the expression of the gene that encodes the chemokine ligand CXCL13. In general, there is no particular trend with respect to microglial polarization, although under the conditions of the A_3_R activation assay here performed, increases in the expression of genes known to be involved in M2 polarization are not obtained (with the exception of PPAR-γ).

## Discussion

**Fig. 4:**
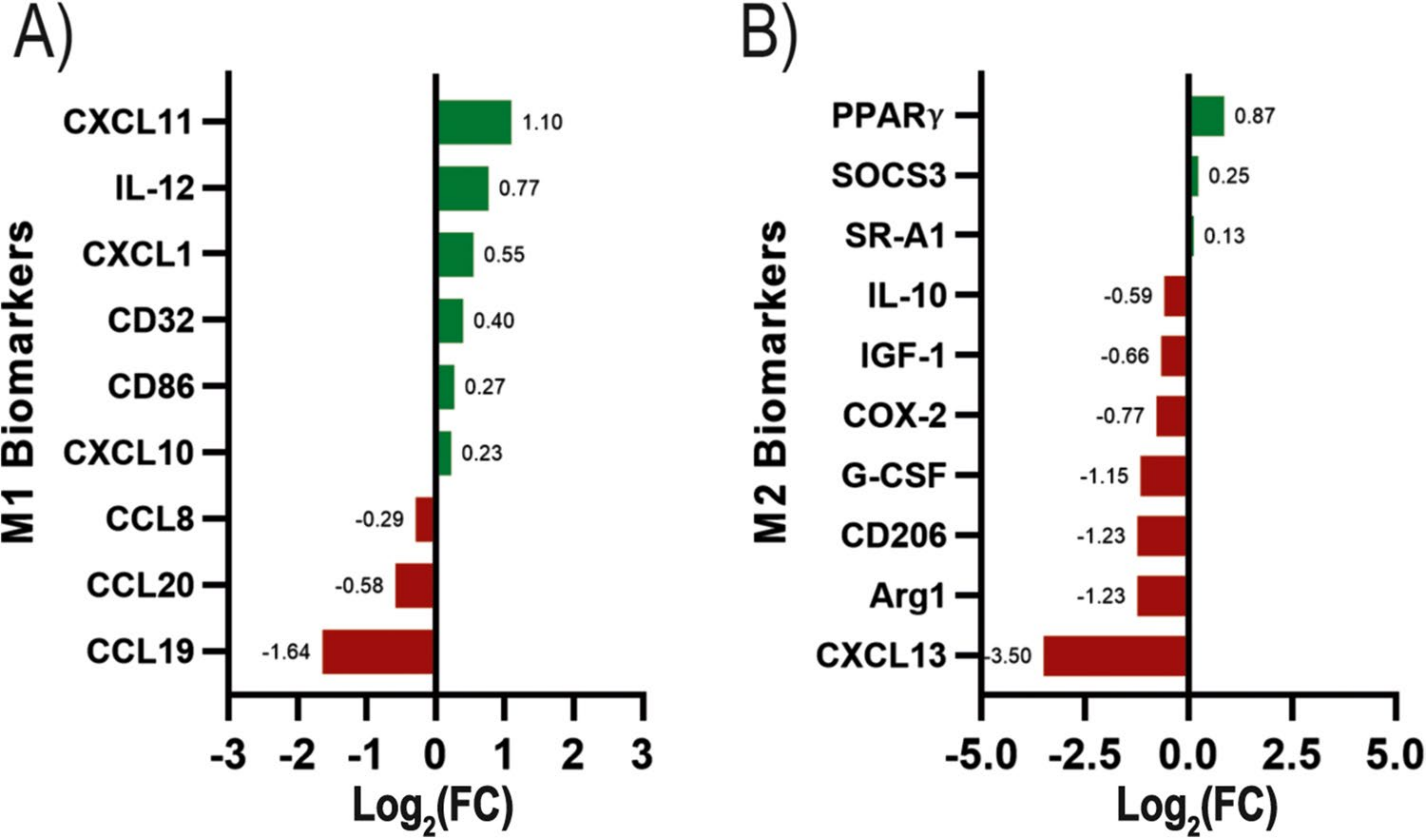
Histogram showing the relative expression of genes for microglial phenotype-related biomarkers. **A** Degree of variation (log_2_FoldChange-FC-) of genes for M1 biomarkers. **B** Degree of variation (log_2_FoldChange) of genes for M2 biomarkers. Increases in expression due to agonist treatment are in green, and decreases are in red. For all these data, the FDR was < 0.05; that is, genes whose expression was not significantly altered upon A_3_R treatment are not shown. Only genes relevant to M1/M2 polarization are shown

The transcriptomics data here presented confirms both A_3_R expression in activated microglia and a relevant role of the receptor in mediating the regulation of microglial activation by adenosine. One of the relevant findings is the large number of genes whose expression is altered by the activation of A_3_R. The number of genes in the mouse genome with protein sequence data is reportedly 25,059 [22]; therefore, the number of genes whose expression is significantly regulated by 2-Cl-IB-MECA was about 8% of the total. A limitation of the study is the assay conditions, namely, robust activation using both LPF and IFN- γ, and 3 doses of the agonist at 24, 32, and 40 h after initiation of activation. Our results are in contrast to the reported beneficial effects of 2-Cl-IB-MECA after subarachnoid hemorrhage in aged rats. The anti-inflammatory effects in this study are seemingly mediated by the P38/STAT6 pathway; however, details on the nature of microglial cells are lacking [21]. Differential activation and/or different agonist treatment protocols could lead to quantitative and/or qualitative differences in results. An example is the reduced expression of inducible nitric oxide synthase (iNOS) by 2-Cl-IB-MECA in retinal microglia under elevated hydrostatic pressure mimicking elevated intraocular pressure, that is, glaucomatous conditions [23, 24]. The BV2 microglial cell line treated with LPS expresses the A_3_R whose activation suppresses TNF-α production by a mechanism that depends on Akt and PI 3-kinase [25]. In a report by Koscsó et al. [26], activation of the A_2B_ receptor in microglia leads to increase in IL10 production, whereas activation of the A_3_R leads to downregulating the expression of the gene coding for IL10 (Fig. 4). Interestingly, the mechanisms mediated by A_2B_ receptor activation depends on Creb [26], a gene whose expression was found to be regulated upon A_3_R activation (Fig. 3).

A second relevant finding was that increases in gene expression occurred much less than decreases in gene expression. It is reasonable to hypothesize that adenosine, via A_3_R, is moderating microglial activation, thus reducing the burden associated to keeping a high degree of gene expression. Transcription factors whose gene expression was regulated by 2-Cl-IB-MECA in activated microglia (Fig. 2, Table 1) are involved in various cellular functions, from the most general to the most specifically related to regulation of immunological responses. Gene ontologies detected after data analysis showed significant clustering with developmental processes, and this fits with the finding that, in microglia, A_3_R agonists promote ADP-induced process extension and migration [27].

The M0 for resting, M1 for pro-inflammatory, and M2 for homeostasis/restoration nomenclature was first coined for macrophages (see [28]), cells that in the periphery perform a function equivalent to that of microglia in the CNS. It is considered that adenosine receptors in macrophages have relevant regulatory functions, thus being potential targets in the therapy of inflammatory diseases (see [29, 30] for review). As in microglia, A_3_R activation in macrophages inhibits TNF-α production [31–33] and increases the LPS-stimulated IL-10 production [33]. Agonists of the receptor exert anti-inflammatory effects by reducing the production of macrophage inflammatory protein-1α (MIP-1α) in immunostimulated RAW macrophages [34]. In summary, in both microglia and macrophages, the activation of A_3_R presumably leads to anti-inflammatory actions.

The transcriptomics study presented here does not provide a basis for a M2 polarization unless there are M2 biomarkers that are still unknown. In regard to M1 polarization, the results are conflicting as there are some genes that are upregulated and others that are downregulated. This fact could indicate that there are intermediate phenotypes [35, 36]. The M0/M1/M2 nomenclature has been instrumental in pointing out that microglial activation can progress to neuroprotective phenotypes [16], but the actual phenotypes may not be clearly established based on M1/M2 markers. Transcriptomics studies may help to elucidate differential activation modes, for instance, comparing results coming from cells activated in a more physiological way. To assess the potential of A_3_R in microglia as a target for neuroprotection, it is also important to play with the doses and find the right time to act, neither too early nor too late. Also relevant is to consider pathways that have not been previously considered. Our results suggest that for better assessing both microglial activation and the potential of A_3_R agonists in neuroprotection, Creb1, and Smad3 and the RFX transcription factor family must be considered.

### Supplementary Information

Supplementary Table S1. Gene expression in activated microglia treated with 2-Cl-IB-MECA compared with untreated activated microglia (see supplementary file) (XLSX 799 kb)

## References

[1] Serrano-Marín J, Reyes-Resina I, Martínez-Pinilla E et al (2020) Natural compounds as guides for the discovery of drugs targeting G-protein-coupled receptors. Molecules 25. 10.3390/MOLECULES25215060 10.3390/molecules25215060 PMC7663367 33143389

[2] Manalo RVM, Medina PMB (2018) Caffeine protects dopaminergic neurons from dopamine-induced neurodegeneration via synergistic adenosine-dopamine D2-like receptor interactions in transgenic caenorhabditis elegans. Front Neurosci 12. 10.3389/FNINS.2018.00137 10.3389/fnins.2018.00137 PMC5845907 29563862

[3] Madeira MH, Rashid K, Ambrósio AF (2018). Blockade of microglial adenosine A2A receptor impacts inflammatory mechanisms, reduces ARPE-19 cell dysfunction and prevents photoreceptor loss in vitro. Sci Rep.

[4] Borea PA, Gessi S, Merighi S, Varani K (2016). Adenosine as a multi-signalling guardian angel in human diseases: when, where and how does it exert its protective effects?. Trends Pharmacol Sci.

[5] Sitkovsky MV, Hatfield S, Abbott R (2014). Hostile, hypoxia-A2-adenosinergic tumor biology as the next barrier to overcome for tumor immunologists. Cancer Immunol Res.

[6] Gao ZG, Jacobson KA (2019) A2b adenosine receptor and cancer. Int J Mol Sci 20. 10.3390/ijms20205139 10.3390/ijms20205139 PMC6829478 31627281

[7] Bar-Yehuda S, Barer F, Volfsson L, Fishman P (2001). Resistance of muscle to tumor metastases: a role for A3 adenosine receptor agonists. Neoplasia.

[8] Yan L, Burbiel JC, Maaß A, Müller CE (2003). Adenosine receptor agonists: From basic medicinal chemistry to clinical development. Expert Opin Emerg Drugs.

[9] Gnad T, Scheibler S, Von Kugelgen I (2014). Adenosine activates brown adipose tissue and recruits beige adipocytes via A2A receptors. Nature.

[10] Gnad T, Navarro G, Lahesmaa M et al (2020) Adenosine/A2B receptor signaling ameliorates the effects of aging and counteracts obesity. Cell Metab 32. 10.1016/j.cmet.2020.06.006 10.1016/j.cmet.2020.06.006 PMC7437516 32589947

[11] Ramirez AI, de Hoz R, Salobrar-Garcia E (2017). The role of microglia in retinal neurodegeneration: Alzheimer’s disease, Parkinson, and glaucoma. Front Aging Neurosci.

[12] Hickman S, Izzy S, Sen P (2018). Microglia in neurodegeneration. Nat Neurosci.

[13] Simões AP, Silva CG, Marques JM (2018). Glutamate-induced and NMDA receptor-mediated neurodegeneration entails P2Y1 receptor activation. Cell Death Dis.

[14] Joshi AU, Minhas PS, Liddelow SA (2019). Fragmented mitochondria released from microglia trigger A1 astrocytic response and propagate inflammatory neurodegeneration. Nat Neurosci.

[15] Calvo-Rodriguez M, Hou SS, Snyder AC (2020). Increased mitochondrial calcium levels associated with neuronal death in a mouse model of Alzheimer’s disease. Nat Commun.

[16] Franco R, Fernández-Suárez D (2015). Alternatively activated microglia and macrophages in the central nervous system. Prog Neurobiol.

[17] Navarro G, Borroto-Escuela D, Angelats E (2018). Receptor-heteromer mediated regulation of endocannabinoid signaling in activated microglia. Role of CB1 and CB2 receptors and relevance for Alzheimer’s disease and levodopa-induced dyskinesia. Brain Behav Immun.

[18] Jacobson KA, Merighi S, Varani K (2018). A 3 adenosine receptors as modulators of inflammation: from medicinal chemistry to therapy. Med Res Rev.

[19] Galvao J, Elvas F, Martins T (2015). Adenosine A3 receptor activation is neuroprotective against retinal neurodegeneration. Exp Eye Res.

[20] Choi I-Y, Lee J-C, Ju C (2011). A3 adenosine receptor agonist reduces brain ischemic injury and inhibits inflammatory cell migration in rats. Am J Pathol.

[21] Li P, Li X, Deng P (2020). Activation of adenosine A3 receptor reduces early brain injury by alleviating neuroinflammation after subarachnoid hemorrhage in elderly rats. Aging.

[22] Bult CJ, Eppig JT, Blake JA (2016). Mouse genome database 2016. Nucleic Acids Res.

[23] Rodrigues-Neves AC, Aires ID, Vindeirinho J (2018). Elevated pressure changes the purinergic system of microglial cells. Front Pharmacol.

[24] Ferreira-Silva J, Aires ID, Boia R (2020). Activation of adenosine A 3 receptor inhibits microglia reactivity elicited by elevated pressure. Int J Mol Sci.

[25] Lee JY, Jhun BS, Oh YT (2006). Activation of adenosine A3 receptor suppresses lipopolysaccharide-induced TNF-α production through inhibition of PI 3-kinase/Akt and NF-κB activation in murine BV2 microglial cells. Neurosci Lett.

[26] Koscsó B, Csóka B, Selmeczy Z (2012). Adenosine augments IL-10 production by microglial cells through an A2B adenosine receptor-mediated process. J Immunol.

[27] Ohsawa K, Sanagi T, Nakamura Y (2012). Adenosine A3 receptor is involved in ADP-induced microglial process extension and migration. J Neurochem.

[28] Mills CD (2012). M1 and M2 macrophages: oracles of health and disease. Crit Rev Immunol.

[29] Haskó G, Pacher P, Deitch EA, Vizi ES (2007). Shaping of monocyte and macrophage function by adenosine receptors. Pharmacol Ther.

[30] Haskó G, Pacher P (2012). Regulation of macrophage function by adenosine. Arterioscler Thromb Vasc Biol.

[31] Sajjadi FG, Takabayashi K, Foster AC (1996). Inhibition of TNF-alpha expression by adenosine: role of A3 adenosine receptors. J Immunol.

[32] McWhinney CD, Dudley MW, Bowlin TL (1996). Activation of adenosine A3 receptors on macrophages inhibits tumor necrosis factor-alpha. Eur J Pharmacol.

[33] Haskó G, Szabó C, Németh ZH (1996). Adenosine receptor agonists differentially regulate IL-10, TNF-alpha, and nitric oxide production in RAW 264.7 macrophages and in endotoxemic mice. J Immunol.

[34] Szabó C, Scott GS, Virág L (1998). Suppression of macrophage inflammatory protein (MIP)-1alpha production and collagen-induced arthritis by adenosine receptor agonists. Br J Pharmacol.

[35] Mesquida-Veny F, Del Río JA, Hervera A (2021). Macrophagic and microglial complexity after neuronal injury. Prog Neurobiol.

[36] Walker DG, Lue L-F (2015). Immune phenotypes of microglia in human neurodegenerative disease: challenges to detecting microglial polarization in human brains. Alzheimers Res Ther.

